# Fan-out Estimation in Spin-based Quantum Computer Scale-up

**DOI:** 10.1038/s41598-017-13308-0

**Published:** 2017-10-17

**Authors:** Thien Nguyen, Charles D. Hill, Lloyd C. L. Hollenberg, Matthew R. James

**Affiliations:** 1 Research School of Engineering, College of Engineering and Computer Science, The Australian National University, Canberra, ACT 2601 Australia; 2 ARC Centre for Quantum Computation and Communication Technology, School of Physics, University of Melbourne, Victoria, 3010 Australia; 3 ARC Centre for Quantum Computation and Communication Technology, Research School of Engineering, College of Engineering and Computer Science, The Australian National University, Canberra, ACT 2601 Australia

## Abstract

Solid-state spin-based qubits offer good prospects for scaling based on their long coherence times and nexus to large-scale electronic scale-up technologies. However, high-threshold quantum error correction requires a two-dimensional qubit array operating in parallel, posing significant challenges in fabrication and control. While architectures incorporating distributed quantum control meet this challenge head-on, most designs rely on individual control and readout of all qubits with high gate densities. We analysed the fan-out routing overhead of a dedicated control line architecture, basing the analysis on a generalised solid-state spin qubit platform parameterised to encompass Coulomb confined (e.g. donor based spin qubits) or electrostatically confined (e.g. quantum dot based spin qubits) implementations. The spatial scalability under this model is estimated using standard electronic routing methods and present-day fabrication constraints. Based on reasonable assumptions for qubit control and readout we estimate 10^2^–10^5^ physical qubits, depending on the quantum interconnect implementation, can be integrated and fanned-out independently. Assuming relatively long control-free interconnects the scalability can be extended. Ultimately, the universal quantum computation may necessitate a much higher number of integrated qubits, indicating that higher dimensional electronics fabrication and/or multiplexed distributed control and readout schemes may be the preferredstrategy for large-scale implementation.

## Introduction

Building a large-scale quantum computer which can solve classically intractable problems is a technologically daunting task. With their close connection to highly scalable classical electronics^1^ solid-state spin qubit platforms, such as donor-based qubits^2–10^ and quantum dots^9,11–15^, are emerging as promising candidates^16,17^, for scalable quantum computation. On semiconducting materials, e.g. Si, SiGe, or GaAs, it is possible in principle to fabricate a large number of interconnecting qubits for quantum information processing. However, in designing such a large-scale solid-state quantum chip, there is still a gap between the quantum computer architecture^18–26^ and the physical qubit device implementation^9,11,12^, Architectures necessarily must incorporate fault-tolerant quantum error correction in order to perform quantum algorithms^27^ at the logical quantum gate level. The physical implementation generally deals with individual qubits on the basis of physical quantum gate operations, initialisation, and readout which are the foundation for higher level quantum logical operations. In the middle ground, quantum computer micro-architectures^25,26,28^, attempt to bridge that gap by providing engineering solutions to issues such as classical control, fan-out interconnects, and chip layout.

Since the quantum error correction protocol is the major contributor to the escalating number of qubits required for quantum computation, finding an optimal error correction code is crucial for a scalable quantum computing architecture. The most well-known figure-of-merit of any quantum error correction code is the *error threshold* which is the bound of physical error rate that the code can tolerate for effective scaling. In this regard, among all currently-known error correction codes, the surface code^29–32^ has been proven to be one of the best-performing codes with the threshold around 1%^31,33,34^.

One key advantage of the surface code is its nearest-neighbor interaction scheme which scales favorably over the concatenation approach. However, this scheme also requires a two-dimensional qubit layout and parallel control. In terms of micro-architecture considerations, one must account for (a) the spatial/geometrical requirements of a 2D nearest-neighbor interacting qubit array, and (b) the temporal/control requirements of parallel/synchronous QEC operations. Broadly, one can identify two approaches. In the ubiquitous independent control model, each quantum element (qubit, gate, interconnect, readout) are controlled independently. In principle, this approach has the highest density of quantum control gates each of which must be carefully characterised and timed to allow for parallel operation across the qubit array (in a number of steps which does not depend on the array size). At the other extreme, in the distributed control model introduced in Hill *et al*.^26^, a high degree of multiplexing allows sufficiently large groups of qubits to be controlled and readout with the required parallelism.

Some authors have attempted to address the problem in the independent control approach by assuming the qubit lattice can be broken into smaller sparsely linked 2D arrays^35^, however, such tiling schemes in general present significant difficulties in implementing the full range of logical operations required by the surface code. We instead focus on the spatial/geometrical challenge of fabricating and scaling up of the full monolithic surface code under the assumption of the independent (non-distributed) control model in order to compare with the distributed control scheme. Quantum interconnect protocols to reduce the qubit density are encapsulated in our study by adding extra coupling surface gates which drive the transport protocols, and assuming operational errors can be accommodated in the QEC protocol. In terms of gate density, the generalised quantum interconnect model effectively captures most interconnect mechanisms by adjusting the number of control gates per interconnect channel.

Under our generalised independent control model, we apply known techniques in interconnect routing to analyse the geometrical scaling problem of surface code control fan-out. We consider two types of solid-state spin qubits: atomically confined qubits (such as phosphorus donors in silicon)^2–4,6,7,10^, and electrostatically confined quantum dot qubits^9,11–15^. In the non-distributed independent control approach, every qubit on the surface code lattice has its own separate control and readout structures that need to be fanned out. The qubit geometry is parametrised by a universal unit cell which can be used to represent both donor-based and quantum dot implementations including the quantum interconnects to neighboring cells by adjusting the number of gates in the unit cell. Other dimensional parameters are selected based on experimental and technological considerations. We must also stress that the scalability of 2D spin qubit arrays depends on multiple factors, not just the control fan-out which we study in this paper. In particular, one must also address the various control issues such as parallelizability, synchronisation, control characterisation, and cross-talk as well as the overall thermal budget given the system will be required to operate at cryogenic temperatures.

## Surface Code Error Correction

**Figure 1:**
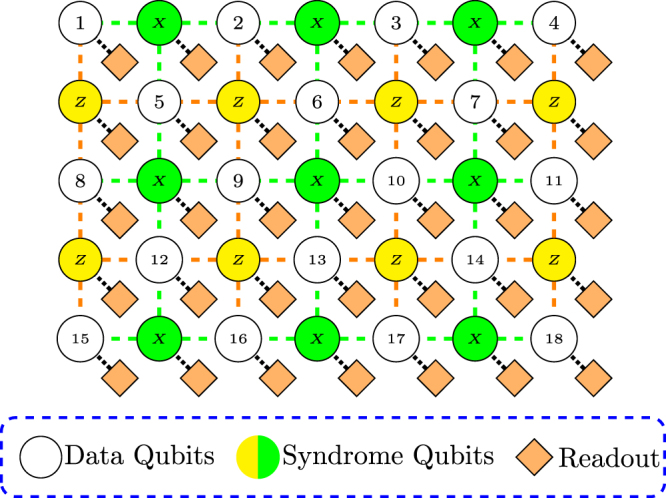
Diagram of surface code lattice with embedded readout devices. There are two types of qubits: data qubits and syndrome qubits (*X* and *Z* types). Neighboring qubits can interact with each other in order to perform CNOT gates. In this model, each qubit has its own readout device. Dashed lines (black) represent quantum interconnects between neighboring qubits. Dotted lines indicate qubits to which readout devices are associated.

Among a wide variety of quantum error correction codes, the *surface code* ^30,32–34,36^, has stood out in terms of computational error threshold which is about two orders of magnitude higher than that of conventional concatenated coding schemes. For the purposes of this paper, the important feature is that implementing the surface code requires a regular 2-D arrangement of qubits, where neighboring qubits interact with each other in a pairwise manner and in parallel (see Fig. 1). Qubits are classified either as *data* qubits or *syndrome* (*ancilla*) qubits according to their roles in the quantum error correction procedure. Each syndrome qubit measurement fixes an eigensubspace of a *stabilizer* operator, which involves all four neighboring data qubits. Logical qubits are defined as topological *defects* on the qubit lattice where syndromes are not measured. Thus, there are two types of logical qubits, so-called *smooth* (*Z*-cut) and *rough* (*X*-cut) logical qubits. The code *distance* is defined either by the perimeter of the defects or the distance between them, whichever is smaller. Interested readers should consult Fowler *et al*.^32^ for an in-depth review.

## Solid-State Spin Qubit Unit Cell Model

In this analysis, we will consider solid-state spin-based quantum computer platforms with a model that encompasses both donor qubits and quantum dot qubits. To construct a basic unit cell model for the micro-architectural fan-out routing analysis, the low-level physics of the quantum devices, as well as high-level quantum computing architecture, can be abstracted by making the following three assumptions:

- Generalised quantum spin interconnects between neighboring qubits,
- Dedicated single-shot spin readout for every qubit,
- Single-sided metallization routing.
- Uniform interconnect dimension and spacing.

The first assumption regards the mechanism by which the qubit-qubit interaction is realised. In principle, we could only implement direct spin-spin coupling, e.g. by spin exchange or dipole couplings, however, direct spin couplings require stringent spacing between qubits which restricts the control and readout routing. By adding interconnects between qubits, we have some flexibility in arranging the qubits and thus can analyse the fanout scalability accordingly. Secondly, we assume each qubit in the array has its own spin read-out device which is usually a Single Electron Transistor (SET^37^) or equivalent^38–42^. This assumption may appear to be more than necessary since neighboring qubits can share a common readout device by using some forms of readout multiplexing, for example, the schemes presented in ref.^26,43,44^, However, for our generic fan-out analysis, this serves as a baseline scenario from which we can straightforwardly adapt to other cases by modifying the gate count per qubit to reflect other specific configurations with readout multiplexing. We assume that the metal routing layers are built on a single side of the substrate. This is the predominant routing technology used by the semiconductor industry. Lastly, we assume that the feature size of interconnect wires is consistent between metallization layers. A pictorial representation of the qubit array structure with dedicated readout devices is shown in Fig. 1.

Regarding the interconnect protocols, while there seems to be a plethora of coherent spin transport/coupling mechanisms^44–53^, for the purpose of our fanout analysis, the main factor to consider is the number of additional control gates versus interconnect length. We, therefore, consider two broad categories: (i) gate count grows linearly with the interconnect length, and (ii) gate count is fixed and independent of the interconnect length. For instance, SWAP-based interconnect and spin shuttling protocols^44,54^, belong to the first category since we need surface gates along the channel to execute the quantum operations for spin swapping or shuttling. On the other hand, protocols such as CTAP^20,47,55^, capacitive coupling via floating gate^52^, spin chain^45,46,48^, microwave line coupling^56–58^, electric dipole coupling^53^, and surface acoustic wave spin transport^49–51^, are some examples of the second category because in those protocols we only need to have some additional transport control gates at the ends of the interconnect not along the channel. In what follows, we will use the terms *spin shuttling interconnect* (SSI) and *end control interconnect* (ECI) for those two interconnect categories, respectively. The overall length of the interconnect is *L* (for interconnect schemes based on qubit chains we equivalently describe the interconnect length in terms of the number of nodes, *N*_n*odes*_).

**Figure 2:**
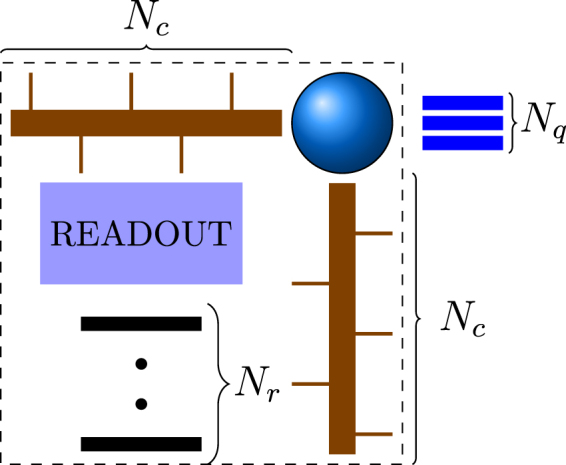
Diagram of a generic surface code array unit cell. Each qubit (circle) has a certain number of surface gates (*N*_*q*_) to define qubit confinement potential and to perform single-qubit rotations. Between any pair of neighboring qubits, we have *N*_*c*_ coupling gates that are used to control qubit interconnect coupling. At the center of the cell, we have a readout device that has *N*_*r*_ surface gates.

In terms of physical qubit implementation, we categorise the surface metal gates that need to be fanned out for controllability and readout into three categories: qubit confining and control (*N*_*q*_), interconnect coupling control (*N*_*c*_), and readout (*N*_*r*_). The types of physical spin qubits considered are primarily classified by the confinement mechanism, i.e. either via an atomic Coulomb potential (e.g. donors) for which we assume *N*_*q*_ = 1, or via electrostatic gates (e.g. quantum dots), for which we assume *N*_*q*_ = 3. Since we assume spin coupling based interconnects, the center-to-center distance (pitch) between qubits needs to be sufficiently small. We use the qubit-qubit pitch of 20 nm and 50 nm for Coulomb-confined and electrostatically-confined qubits, respectively. Using the above gate classification, the surface code lattice can be decomposed into unit cells, each of which contains one qubit and one readout device as shown in Fig. 2. When partitioning the surface code lattice as shown in Fig. 1, there are four equivalent interactions, namely along the north-east, north-west, south-east, or south-west direction. For example, the unit cell in Fig. 2 is a south west participation scheme where the interconnects and readout device on the bottom left of a qubit are associated with that qubit for analysis purposes.

As indicated, we will categorise the interconnect protocols into two groups: SSI, where the number of interconnect control gate count grows linearly with interconnect length and ECI with a fixed number of interconnect surface gates regardless of interconnect length. The gate count assumptions for these two scenarios are listed in Table 1. The surface contacts are placed directly on top of the qubits, interconnect rails and readout devices. In order to facilitate routing, these gate contacts are then redistributed into a square grid array. The surface code qubit array can then be assembled by placing unit cells next to each other, thus forms a regular global square grid array used for fan-out routing.

**Table 1:** Gate count configurations for spin shuttling interconnect (SSI) and end control interconnect (ECI) protocols.

Interconnect type	SSI	ECI
N_c_	4 × *N*_nodes_	4
N_r_	3	3

The interconnect node count (*N*_nodes_) is the number of intermediate qubit nodes along the interconnect channel.

It is worth noting that qubits (dots or donors) along the interconnect rails in Fig. 2 are not counted as physical qubits in the following analyses. Only the corner qubit of the unit cell which can act as a data or syndrome qubit in the surface code (Fig. 1) is accounted for as a physical qubit in the scalability study. In fact, several ECI schemes that we mentioned earlier do not require intermediate qubit nodes at all, e.g. microwave or capacitive coupling. For this scheme, the absolute interconnect distance is the only relevant parameter.

For ECI and SSI schemes that involve qubit chains, an important issue may arise which is the loss of qubits during transfer/coupling (due to operational errors or permanent manufacturing defects). While acknowledging that there are quantum error correction methods and techniques^59–63^, to mitigate qubit loss, this aspect of qubit connectivity is outside the scope of our considerations here. Therefore, we assume the feasibility of reliable quantum interconnects in order to focus on the issue of fan-out routing scalability.

The contact pitch after redistribution is related to the interconnect length (*L*) by the following inequality:

1 $${p}_{{\rm{RL}}}\le \frac{{\rm{unit}}\,{\rm{cell}}\,\mathrm{dimension}({\rm{L}})}{\sqrt{{N}_{{\rm{total}}}({L})}},$$

where *N*total is the total number of gate contacts in a unit cell and *p*_*RL*_ is the contact pitch at the redistribution layer (RL). This total gate count may or may not depend on the interconnect length. On the other hand, the unit cell dimension is proportional to the interconnect length *L*. We can clearly see that by increasing the length of the interconnect (*L*), the contact pitch after redistribution is extended since the denominator is either constant or growing on the scale of square root of *L* while the nominator grows linearly with *L*. In principle, larger pitches will benefit the global fan-out routing as more interconnect routing space is created. This is explained in the Methods section where we describe the routing parameters and the two commonly-used fan-out routing algorithms.

At the redistribution layer the dimension parameters are *d* = 10 nm, *w* = 5 nm, and *s* = 25 nm whilst the contact-contact pitch equals to the redistributed pitch computed by (1). However, there is a minimum contact-contact pitch which needs to be satisfied, namely *p*_min_ = *d* + *s* = 35 nm. Thus, there is a lower bound on the interconnect length to space the contacts sufficiently according to (1). The worst-case scenario occurs in the SSI scheme for Coulomb-confined qubits because of their tight qubit-qubit spacing and increasing number of coupling gates with interconnect length. We can estimate the minimum interconnect length by using equation (1) in conjunction with the gate count data in Table 1 and the qubit pitch assumption, e.g. for the 20 nm case we have

2 $${p}_{{\rm{RL}}}\approx \frac{{\rm{20}}\,{\rm{nm}}\times {N}_{{\rm{nodes}}}}{\sqrt{1+4{N}_{{\rm{nodes}}}+3}} > {\rm{35}}\,{\rm{nm}},$$

which requires a minimum interconnect length (*min*(*N*_nodes_)) of 14 nodes (280 nm). Following the same procedure, we can derive the minimum interconnect length for all configurations in terms of *N*_*q*_ configurations and interconnect schemes as shown in Table 2.

**Table 2:** Minimum interconnect length in terms of chain node-count and absolute distance for spin shuttling interconnect (SSI) and end control interconnect (ECI) protocols.

*min*(*N*_nodes_)/*L*_interconnect_	SSI	ECI
*N*_*q*_ = 1 (20 nm)	14/280 nm	5/100 nm
*N*_*q*_ = 3 (50 nm)	3/150 nm	3/150 nm

Qubit-qubit pitch is 20 nm for atomically confined qubit (*N*_*q*_ = 1) and 50 nm for electrostatically confined qubit (*N*_*q*_ = 3).

## Results

**Figure 3:**
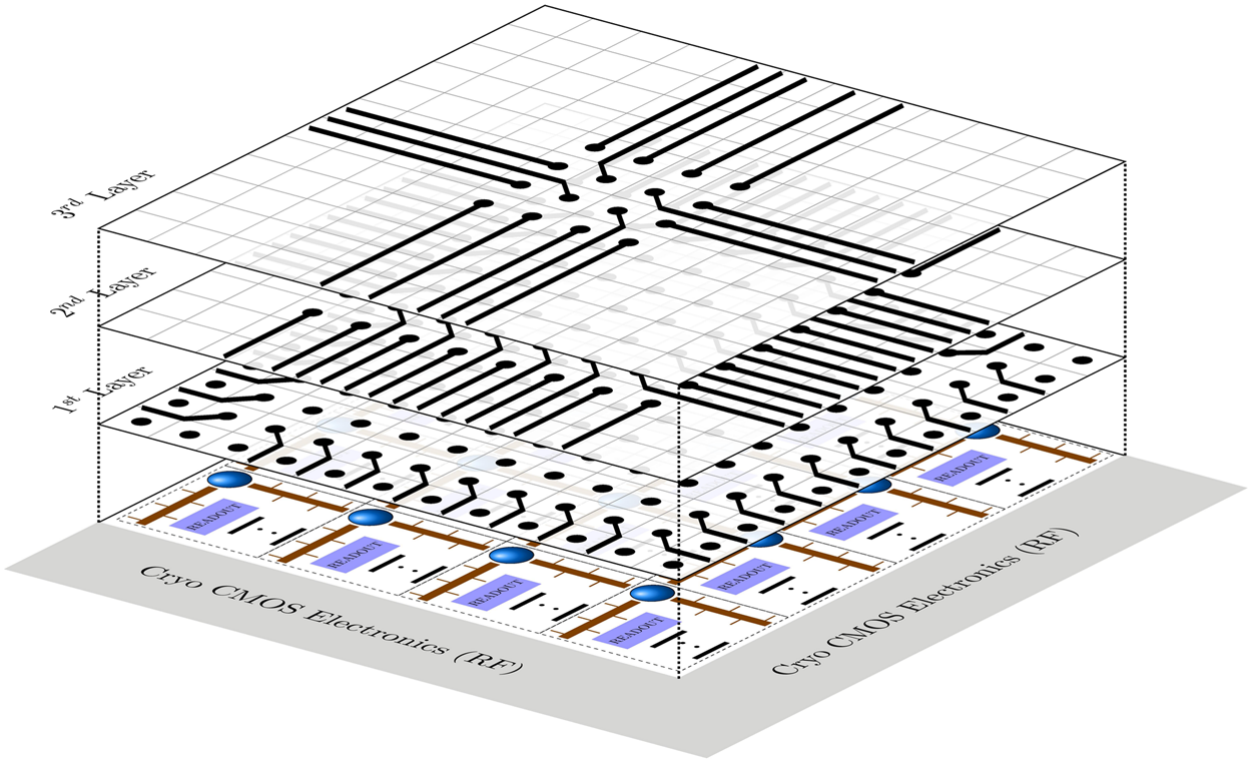
Illustration of 2-D qubit lattice surface gate fanout using multiple metal routing layers. The bottom layer is a semiconductor material (Si or GaAs) with top gates for control and readout. On the same substrate lies classical integrated electronics used for signal generation, multiplexing, and sensing. In order to bring connections to the surface gates, multi-layer routing is needed. After surface gates are redistributed into a square-grid array of contacts, as shown in the first metal layer, the fan-out routing procedure is carried out layer-by-layer using a specific routing algorithm. This figure demonstrates ring-by-ring routing, which requires three metal layers for this particular grid array. More sophisticated routing algorithms can be implemented using EDA (Electronic Design Automation) tools.

Generally, in order to supply the electrical signals to the control gates or the readout devices to perform qubit readout, each and every gate needs to be fanned out to connect to the classical control systems. In conventional nanoelectronics, this is achieved by overlaying the qubit array with many metal lines on several layers. Electrical connections from these metal lines to the surface gates are made by vertical conducting “vias”. The unique advantage of Si-based solid-state quantum platforms is the compatibility with the classical CMOS electronics, whereby both can be integrated onto the same silicon chip. Classical electronics can be fabricated outside the surface code qubit lattice as shown in Fig. 3. At the bottom layer lies the semiconducting material substrate in which qubits are realised and controlled by surface gates. Therefore, we need to fan the surface gates out to the peripheral classical electronics area where classical processing tasks are performed. Under the generic model considered here, regardless of the interconnect protocols, the gate contacts/vias are redistributed into a square-grid array before global fan-out routing is performed, as illustrated by metal routing layers shown in Fig. 3.

The fan-out scalability of 2D qubit arrays is examined by looking at the number of routing layers required for complete routability. As shown in Fig. 3, multi-layer routing can potentially provide unlimited fan-out capacity if we let the number of metal layers be unbounded. However, in practice it is imperative to keep the number of metallization layers to the absolute minimum - usually in the range of 10–15 layers for the most advanced semiconductor products^64^. The technological and economic challenges associated with fabricating many layers of nano-scale interconnects are going to be similar for the various solid-state quantum computing approaches. In the following analyses, we stretch to a 20 routing layer limit to benchmark the fan-out scalability of the various quantum interconnect schemes. We will adopt two standard routing algorithms from classical electronics, namely the ring-by-ring and the layer optimisation algorithms, which are described in detail in the Methods section.

**Figure 4:**
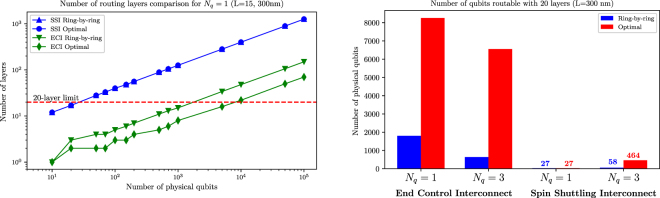
Comparison between different interconnect protocols and routing methods at fixed interconnect length: (left) Plot of the number of routing layers vs. number of physical qubits for atomically confined qubits (*N*_*q*_ = 1) at *L* = 300 nm; and (right) scalability comparison between electrostatically confined qubits (*N*_*q*_ = 3) and atomically confined qubits (*N*_*q*_ = 1) using the same number of routing layers (20) and interconnect length (*L* = 300 nm, 15 nodes for *N*_*q*_ = 1 and 6 nodes for *N*_*q*_ = 3). Dimension parameters are (see Methods): *d* = 10 nm, *w* = 5 nm, and *s* = 25 nm. The red dashed horizontal line on the left figure represents the technological limit of 20 metal layers that can be fabricated reliably and economically on a semiconductor substrate.

First, we look at the raw differences between the two routing algorithms at a fixed interconnect length. The triangular routing (layer optimisation) algorithm is the most efficient way to fan-out all contacts in terms of the required number of layers (see Methods). This is shown in Fig. 4, where we examine both ring-by-ring and layer optimal routing solutions for the SSI and ECI protocols for *L* = 300 nm (which satisfies the minimum interconnect length (14 nodes, 280 nm) for the atomically-confined SSI scheme). It is worth noting that we use the same interconnect length to compute the fan-out for electrostatically-confined qubits (*N*_*q*_ = 3). Because the qubit-qubit distance is different, the number of qubit nodes in the interconnect chain varies across different qubit configurations in the bar chart comparison (Fig. 4, right).

We observe a factor of 5 to 8 increase in the number of routable qubits by using the optimal router across most of the scenarios (except for SSI, *N*_*q*_ = 1) as shown in the right chart of Fig. 4. This highlights the fact that for large-scale qubit integration the use of design-automation tool suites is important to achieve better routing solutions compared to more intuitive and direct methods such as the ring-by-ring method. One exception is in the case of SSI scheme for atomically-confined qubits (*N*_*q*_ = 1) where both routing methods result in the same number of routable qubits. The reason is that at this interconnect length (*L* = 300 nm), the contact-contact pitch after redistribution (eq. (1)) is barely above the minimum metal-metal pitch requirement, thus no escape routing (wires between pads) is allowed.

Another point which can be seen from Fig. 4 is the advantage of the ECI scheme over its SSI counterpart in terms of fan-out scalability. This naturally stems from the fact that ECI protocols require far fewer surface gates than their SSI counterparts (Table 1). We will later investigate the scaling differences between the two schemes in details by looking at various interconnect lengths. The difference in qubit-qubit spacing manifests itself in the opposite trend observed in Fig. 4 bar chart: while atomically-confined qubits are the clear winner in the ECI scheme, the opposite is true if the shuttling interconnect scheme is assumed. This can be explained by the minimum interconnect length data in Table 2, i.e. while *N*_*q*_ = 1 has shorter minimum interconnect requirement in the ECI scheme, it has a much longer minimum interconnect length in the SSI scheme. The scalability difference in the ECI scheme between *N*_*q*_ = 1 and *N*_*q*_ = 3 is narrowed significantly if we use optimal routing for electrostatically-confined qubits (from 2x to about 25% different). In all the following analyses, we will only consider triangle (layer optimised) router since this will better reflect the realistic engineering solution. To highlight the effect of even more confining gates (e.g. double-dots as qubits), we also perform the analysis for a hypothetical case of *N*_*q*_ = 5.

All the scenarios that we consider so far are homogeneous in the sense that corner qubits and interconnect qubit nodes are of the same type. For SSI scheme, we can implement a hybridisation protocol in which atomically confined qubits are used as surface code physical qubits, while electrostatically confined qubits are utilised for interconnect coupling. By doing this, we can achieve the best of both worlds for the SSI scheme, namely minimising *N*_*q*_ and maximising qubit distance. This approach is only effective for SSI scheme since for ECI the number of interconnect gates is constant.

**Figure 5:**
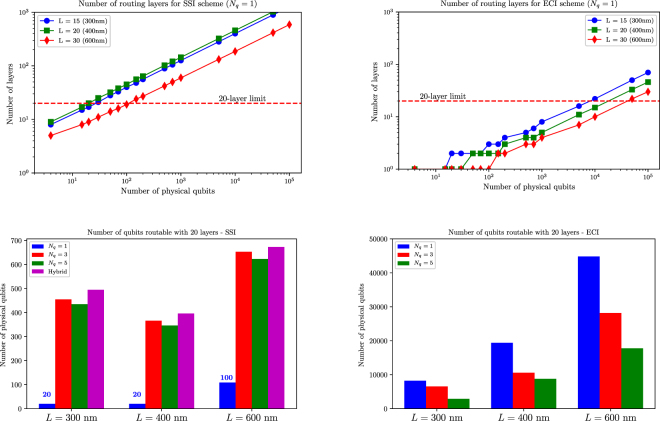
Qubit fanout scalability in the cases of interconnect protocols of (left) SSI and (right) ECI. (Top) Number of routing layers vs. number of physical qubits for atomically confined qubits (*N*_*q*_ = 1) under different interconnect lengths; and (bottom) number of routable qubits comparison between electrostatically confined qubits (*N*_*q*_ = 3 and *N*_q_ = 5), atomically confined qubits (*N*_*q*_ = 1), and hybrid SSI (donors as qubits and dots as shuttling nodes). Dimensional parameters are: *d* = 10 nm, *w* = 5 nm, and *s* = 25 nm. The red dashed horizontal line on top charts represents the technological limit of 20 metal layers that can be fabricated reliably and economically on a solid-state substrate.

Figure 5 shows the fan-out scalability in terms of routing layers (optimised router) for both the SSI and ECI protocols with interconnect length of 300 nm, 450 nm, and 600 nm (15/20/30 nodes and 6/8/12 nodes for *N*_*q*_ = 1 and *N*_*q*_ = 3/5 or SSI hybrid, respectively). In the top graphs, the fan-out scaling of atomically confined qubits (*N*_*q*_ = 1) is analysed in detail to provide a reference and the horizontal line represents the limit of 20 metal layers as previously explained. Other qubit configurations (*N*_*q*_ = 3/5 and SSI hybrid) are compared to this reference in the bottom graphs.

An obvious conclusion which can be drawn from both the left charts in Fig. 5 is that the SSI protocol does not provide a consistent fan-out scaling benefit as compared to its ECI counterpart, as there is no clear trend in terms of the number of routable qubits vs. interconnect length. The main contributing factors to this fluctuating trend are the opposite effects of redistributed pitch extension, the increasing number of gates per unit cell and the granularity of the routing problem (only full routing channels are considered). On the other hand, ECI protocols provide a monotonic improvement in terms of the number of integrated qubits vs. interconnect length because the interconnect length (thus metal pitch) is *N*_*c*_-independent. The order of routability vs. interconnect length for different qubit configurations is reserved for both SSI and ECI schemes. While the former interconnect scheme favours electrostatically-confined qubits due to their long qubit-qubit spacing, the later scheme suits atomically-confined qubits a little bit better thanks to the reduced number of confining gates needed. The hybrid SSI scheme outperforms both of its homogeneous SSI counterparts but noted that the best it can achieve is still an order of magnitude less than that of the ECI scheme.

**Figure 6:**
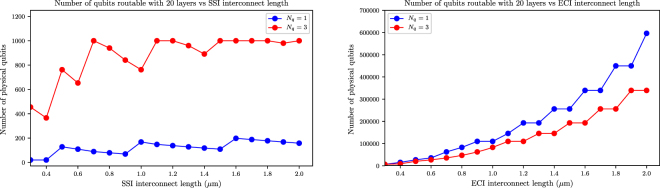
Fan-out scalability vs. interconnect length for (left) SSI and (right) ECI schemes. The inter-qubit interconnect lengths are given in absolute unit (mm). The number of interconnect ubit nodes can be inferred by noting that the qubit-qubit distance is 20 nm for *N*_*q*_ = 1 and 50 nm for *N*_*q*_ = 3.

To assess the fan-out scalability of interconnect protocols over extreme length scale, we extend the interconnect length further (up to 100 intermediate nodes for *N*_*q*_ = 1, i.e. 2 *μ*m). The result is shown in Fig. 6 for the SSI and ECI schemes. This analysis provides a concrete example to the scaling bottleneck of the SSI protocols in 2D qubit lattice implementation (only routable up to about 10^3^ qubits for electrostatically confined qubits and about 200 for atomically confined qubits). The maximum number of routable qubits is saturating over long SSI interconnect length for both types of qubits. Multiplexing schemes for SSI control, e.g. ref.^25,26^ will improve the scalability of these approaches to a certain extent. On the other hand, the ECI protocol can scale up (quadratic) to an order of 10^5^ qubits over that length scale. Again, as we have already seen in Fig. 5, there is an incremental improvement in terms of scalability at the same interconnect length when using electrostatically self-confined qubit structures due to their gate count efficiency. Further steps can be taken to estimate the number of logical qubits feasible based on the level of error correction required, namely the code distance. The latter depends on multiple factors such as the gate fidelity, total number of gates in the algorithm of interest, and the level of output accuracy required. The analysis in ref.^32^ provides estimates of the qubit resource required for surface code quantum computation.

In addition to quantifying the spatial requirements for scale-up, as we have here, there are undoubtedly other aspects which may restrict the scalability of solid-state qubit integration, namely control timing, signal integrity, thermal budget, testability, and manufacturability. Nevertheless, being able to model and extrapolate the limit of each of the scaling bottlenecks is good engineering practice.

## Summary

If the advancement in solid-state spin qubit fabrication and control follows that of their classical counterparts, the number of integrated qubits will soon reach the threshold where scaling up becomes the next bottleneck. As the quantum network gets larger and larger to cope with real-world applications, the amount of routing required to provide control access to surface gates will soon become the limiting factor. By applying the classical electronics know-how regarding interconnect routing to a ubiquitous 2-D qubit array with independent gate control and readout fan-out, we have provided a concrete procedure for scalability estimation, which is adaptable to a wide range of surface code implementations by adjusting the gate configuration and dimensional parameters. This estimation procedure is important for large-scale quantum processor design process where we need to identify at the very early stages the required specifications (so-called “*landing zones*” in classical electronics design) regarding quantum interconnect length and fidelity, back-end metal interconnect dimensions and the number of fan-out layers. For architectures where each qubit has its own dedicated control lines and readout device, we have analysed fan-out scenarios associated with two categories of quantum interconnects, namely spin shuttling interconnects (SSI) and end control interconnects (ECI) with high and low gate densities respectively. Both interconnect models help extend the contacts/vias pitch through redistribution, which potentially aids the fan-out routing procedure. However, SSI protocols result in a poorly scalable situation since the added interconnect control gates outweigh the pitch scaling benefit. On the other hand, ECI protocols provide a more consistent fan-out scaling trend with interconnect length, however, relatively long interconnects (greater than several microns) are required to scale the system to the million qubit level, where issues such as interconnect fidelity, characterisation and operation time, will affect the error rate and surface code error correction performance negatively. Above all, the errors induced in the quantum interconnect must be correctable by the QEC protocol. Multiplexing schemes^44,65^, alleviate the gate density bottleneck to some extent, with fully distributed control schemes^26^ providing scalability without the need for quantum interconnects.

## Methods

**Figure 7:**
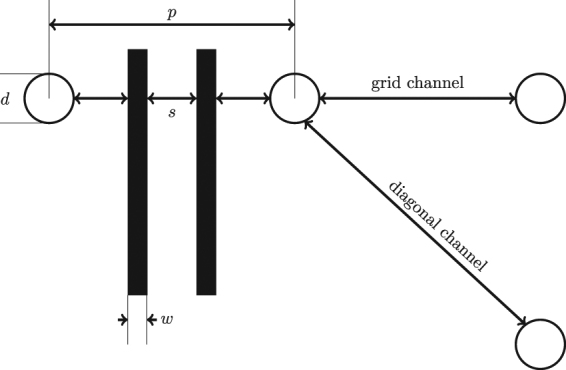
Routing parameters: pad pitch (*p*), wire width and spacing (*w* and *s*), pad diameter (*d*). The grid and diagonal channels are also indicated.

In order to perform the routing analysis, we need to define the geometry of the wiring and via pads. In particular, planar routing on each metal layer depends on the dimension of the metal wires and the spacing between vias. These geometric parameters, which are shown in Fig. 7, can be defined as followings:

- Pitch (*p*): the spacing between two neighboring pads after redistribution
- Pad diameter (*d*): the diameter of the pads
- Line width (*w*): the width of wires
- Line spacing (*s*): the spacing between wires or wires and pads
- Grid channel: the routing space available between two horizontal or vertical pads. Its routing capacity is calculated by:

3 $$C=\lfloor \frac{p-d-s}{w+s}\rfloor $$

- Diagonal channel: the routing space available between diagonal pads. In a square array, its routing capacity is calculated by:

4 $$D=\lfloor \frac{\sqrt{2}p-d-s}{w+s}\rfloor $$

The smaller the wires, the better fanout scaling can be achieved. However, narrow and closely-spaced interconnects also tend to compromise the signal integrity, especially at high frequency. In this work, to provide upper-bound for the scalability, we assume minimal wiring dimension of width(*w*) = 5 nm and spacing(*s*) = 25 nm. Similar nanoscale wires have been fabricated in the lab for nanowire structures^66^. This wiring dimension assumption is also consistent with the International Technology Roadmap for Semiconductors (ITRS) projection that by 2020 mainstream semiconductor manufacturing will reach nanowire diameter of 5 nm. We assume that the via contact diameter will double the wire width, i.e. *d* = 10 nm. Regarding the interconnect pitch (*p*) used for routing, as shown in (1), when we implement longer interconnect chains between qubits, the pitch will be extended.

### Ring-by-ring Routing

A ring-by-ring router will work as follows:

1. Connect the outermost pads directly,
2. Use the grid channels between outermost pads to route internal pads on a ring-by-ring basis,
3. When all the grid channels are exhausted, move up to an upper metal layer and repeat step 1 and 2 until all pads are routed.

**Figure 8:**
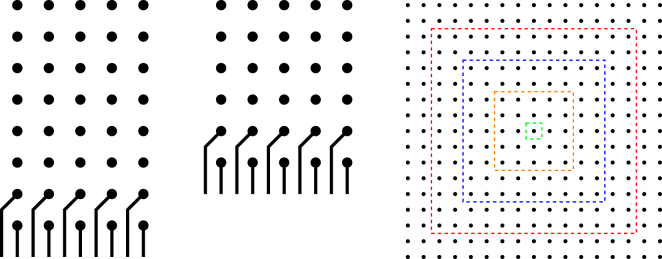
Conventional ring-by-ring routing approach: the outermost ring of unconnected pads are connected first, then inner rings are connected using grid channels of the outer ring until their capacity exhausted. This procedure is then repeated on upper metal layers. The left is the routing on the first metal layer. Similarly, the middle one is the routing on the second layer, and so on. The overall procedure is depicted in the right diagram where each ring denotes the remaining pads after each layer of metallization.

This approach is very intuitive, as shown in Fig. 8. However, the major drawback of this scheme is that its boundaries are quickly shrinking layer-by-layer (as illustrated by the smaller and smaller dotted squares on the rightmost diagram in Fig. 8). Therefore, the routing capacity also decays as we proceed to higher and higher layer. This results in a higher number of metallization layers required as compared to the layer optimisation scheme.

### Layer Optimisation Routing

**Figure 9:**
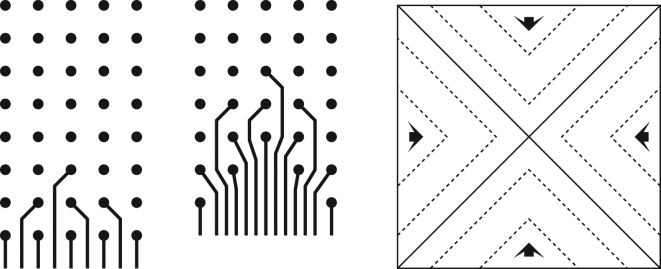
Metal layer optimal routing approach: the routing procedure is performed by proceeding triangularly inward. In this way, it can deploy the diagonal channels, which always have higher routing capacity and take advantage of empty spaces resulted from previously routed pads. The left diagram shows the pads that are routed in the first layer. The middle is the routing on the next layer. The right is the overview of this routing approach.

A second widely used scheme for escape routing is the so-called triangular routing^67^ that is depicted in Fig. 9.

In contrast to the intuitive ring-by-ring approach, this scheme was derived as a maximum flow optimisation problem whereby the opening space left by routed pads in lower layers are utilised maximally, as shown in the middle diagram of Fig. 9. This resulted in a minimal number of layers required to route all the pads.

An *n* × *n* array will require at least *k* layers of routing, where *k* is the smallest integer that satisfies the below inequality^67^:

5 $$-\mathrm{2(}D+\mathrm{1)(}D+\mathrm{2)}{k}^{2}+\mathrm{[4(}D+\mathrm{1)}n-10D+8C]k\ge {n}^{2},$$

where *C* and *D* are the grid and diagonal capacities in Eqs (3) and (4), respectively.

### Data Availability

The datasets generated during and/or analysed during the current study are available from the corresponding author on reasonable request.
